# Chorionic Villi Sampling among Early and Late Gestational Age: Does Timing Affect Yield and Outcomes?

**DOI:** 10.1055/a-2837-7068

**Published:** 2026-04-07

**Authors:** Julia Kim, Amberly Lao, Annie Rozenblyum, Lamia Alamri, Abigail Ludwigson, Teresa Dunn, Andrei Rebarber, Jennifer Lam-Rachlin, Manesha Putra, Martin Chavez, Patricia Rekawek, Lakha Prasannan

**Affiliations:** 1 Department of Obstetrics and Gynecology, NYU Langone Health, NYU Langone Hospital—Long Island, NYU Long Island School of Medicine, Mineola, New York, United States; 2 Division of Maternal Fetal Medicine, Department of Obstetrics and Gynecology, University of Colorado, Aurora, Colorado, United States; 3 Department of Obstetrics and Gynecology, Stanford University School of Medicine, Stanford, California, United States; 4 CytoGenX Corp, Medical Genetic Testing Laboratory, Stony Brook, NY, United States; 5 Maternal-Fetal Medicine Division Mount Sinai West, Icahn School of Medicine at Mount Sinai Hospital, Carnegie Imaging for Women PLLC, New York, United States

**Keywords:** 10–14-week scan, chorionic villus sampling, cytogenetic, pregnancy

## Abstract

**Background:**

Chorionic villus sampling (CVS) is a diagnostic procedure that can be performed between 10 ^0/7^ and 13 ^6/7^ weeks to detect genetic abnormalities; however, a majority of providers opt to perform CVS after 11 weeks. This study evaluated the feasibility of CVS performed at varying gestational ages, comparing chorionic villi (CV) yield and procedural outcomes among early, typical, and late procedures.

**Materials and Methods:**

This multicenter retrospective study included patients with CVS categorized as early (10 ^0/7^ –10 ^6/7^ weeks), typical (11 ^0/7^ –13 ^6/7^ weeks), and late CVS (≥14 ^0/7^ weeks). The primary outcome was median CV weight. Secondary outcomes included need for culture, time to microarray results, and a subanalysis of abnormal chromosomal microarray analysis (CMA) results, obstetric, and neonatal outcomes.

**Results:**

Of 719 patients, 8.1% underwent early, 83.2% typical, and 8.8% late CVS. The early cohort had a lower body mass index (BMI). Early CVS was most frequently performed transvaginally and for the indication of prior affected pregnancy, and less likely for abnormal genetic screening or ultrasound findings. Median villi weight did not differ significantly, and 89% of all procedures yielded adequate tissue, defined as ≥5 mg. The time to the microarray result was shortest in the typical group. There were no significant differences in other secondary outcomes of need for culture, number of passes, or procedure-related complication rates. There was no case of limb anomalies.

**Conclusion:**

CVS performed before 11 weeks and after 14 weeks demonstrated comparable microarray outcomes and demonstrate the technical feasibility and diagnostic adequacy of CVS performed outside the typical gestational window. The results also support the availability of early CVS for cytogenetic testing in early pregnancy loss, where management may not allow for direct tissue testing. Prospective studies are warranted to validate these results and refine recommendations for optimal timing of CVS.

## Introduction

Chorionic villus sampling (CVS) is a form of prenatal diagnostic testing that can be performed between 10 ^0/7^ and 13 ^6/7^ weeks' gestation to detect genetic and chromosomal abnormalities. Chorionic villi (CV), projections of the placenta that contain fetal genetic material, cover the surface of the gestational sac and develop during this time frame to sufficiently allow sampling via a transvaginal or transabdominal approach with a low complication rate. 1 2 After this period, the quantity of accessible villi diminishes with consolidation and increasing placental vascularity, making sampling more challenging with a potentially higher risk of bleeding. 3

Conversely, prior to 10 weeks, villi may be insufficiently developed to yield an adequate tissue sample. Early studies also suggested an increased risk of limb anomalies when CVS was performed before 10 weeks. 4 5 6 7 8 CVS can be safely performed at 10 weeks, but most occur after 11 weeks, which was purportedly done to further minimize any residual risks. 8 9 10 This timing also reflects the widespread use of cell-free DNA (cfDNA) screening at 10 weeks, which lowers the number of screen-negative patients who pursue invasive testing. Most studies and protocols define an adequate sample as ≥5 mg, but limited data exist examining yield and diagnostic adequacy outside the conventional time frame. 11 CVS performed at ≥14 weeks may provide diagnostic results earlier than waiting until amniocentesis at 16 weeks, potentially reducing time to decision-making. CVS performed between 10 and 11 weeks also provides an earlier diagnosis, especially for patients with risk factors. 8 9 By assessing tissue adequacy and clinical outcomes, this study aims to inform practice regarding whether CVS can be reliably performed across a wider gestational age range. The objective of this study was to compare the CV yield and procedural time frame.

## Materials and Methods

A multicenter, retrospective cohort study was performed at three academic U.S. centers: NYU Langone Long Island, Carnegie Imaging for Women PLLC (Maternal-Fetal Medicine Division Mount Sinai West), and the University of Colorado. Patients aged ≥18 years with singleton pregnancies who underwent CVS between 2020 and 2024 (2021 and 2024 at NYU and Carnegie; 2020 and 2023 at Colorado) were included. Pregnancies diagnosed with fetal demise prior to CVS were excluded. CVS was performed by Maternal–Fetal Medicine (MFM) specialists (eight at NYU, nine at Carnegie, and seven at Colorado) with 1 to 20 years of clinical experience. Each institution utilized a single cytogenetics laboratory for tissue analysis. Cytogenetic testing consisted of karyotype and/or microarray analysis according to clinical indication. Analyses and turnaround time were limited to cases in which microarray testing was performed.

A retrospective chart review collected demographic data, pregnancy characteristics, procedural variables, and obstetric outcomes. Variables included age, body mass index (BMI), gravidity, parity, race/ethnicity, spontaneous pregnancy or embryo transfer, medical comorbidities, history of genetic abnormalities, dating method, and gestational age at sampling. Multifetal gestation pregnancies were excluded. Patients were categorized as early (10 ^0/7^ –10 ^6/7^ weeks), typical (11 ^0/7^ –13 ^6/7^ weeks), and late CVS (≥14 ^0/7^ weeks). Despite the current practice to consider CVS beginning at 10 weeks, there are disproportionately fewer procedures occurring at this gestational age due to the availability of cfDNA; therefore, <11 weeks was selected to evaluate this interval specifically.

## Institutional Procedural Details

**NYU Langone Long Island:** CVS performed by eight MFM specialists (with five providers performing early CVS and eight providers performing late CVS). Transvaginal and transabdominal approaches utilized a 5.3-Fr Cook catheter attached to a 20-mL syringe, and an 18- or 20-gauge needle with a 20-mL syringe, respectively.

**Carnegie Imaging Mount Sinai West:** Performed by nine MFM specialists (with six providers performing early CVS and nine providers performing late CVS). Transvaginal and transabdominal approaches utilized a 5.3-Fr Cook catheter attached to a 20-mL syringe, and an 18-gauge needle with a 20-mL syringe, respectively.

**University of Colorado:** CVS performed by seven MFM specialists. Transvaginal procedures used a 5.3-Fr Cook catheter attached to a 20-mL syringe; transabdominal procedures used an 18- or 20-gauge needle with a 10-mL syringe. Samples were rinsed in CHANG Amnio media and Hank's Balanced Salt Solution, then visually weighted by two cytogenetics technologists using a photographic analog scale.

The primary outcome was the median CV weight. Secondary outcomes included need for culture, time to microarray result, need for subsequent amniocentesis, termination of pregnancy (TOP), and spontaneous abortion. An additional subanalysis was performed for the abnormal microarray. Obstetric and neonatal outcomes included gestational age at delivery, preterm birth <37 weeks, and a composite of placental insufficiency outcomes (fetal growth restriction, placental abruption, oligohydramnios, and preeclampsia). CVS variables included route of procedure, number of passes, placental location, indication for CVS, and complications (hemorrhage, bleeding, infection, or limb anomalies detected within 2–4 weeks from CVS).

Descriptive statistics were used to characterize the data. Results are presented with means and standard deviations or medians and interquartile ranges, as appropriate. Because villi weight was non-normally distributed, the median was used for analysis. Categorical variables are expressed as frequency and percentage. Comparisons for continuous variables were performed with the one-way analysis of variance (ANOVA) or Kruskal–Wallis test, as appropriate. Categorical variables were examined using the chi-square or Fisher's exact test, as appropriate.

A multivariable linear regression analysis was performed to evaluate the association between CVS timing and obstetrical outcomes, adjusting for the following potential explanatory variables: Maternal age, BMI, parity, race and ethnicity, preexisting medical comorbidities (chronic hypertension, diabetes, thyroid disease, autoimmune disease, cardiac disease, and psychiatric conditions), and indication for CVS. Adjusted odds ratios are presented along with the corresponding 95% confidence intervals. Statistical significance was defined as *p* < 0.05.

## Results

A total of 719 patients were included: Early ( *n* = 58), typical ( *n* = 598), and late ( *n* = 63). The early CVS group had a significantly lower BMI compared with those in the typical and late groups (24.1 ± 4.6 vs. 27.9 ± 6.3 vs. 28.1 ± 7.4 kg/m ^2^ , respectively, *p* < 0.001, Table 1 ). All other baseline maternal characteristics were similar.

**Table 1:** Patient demographics among early, typical, and late gestational ages

	<11 weeks *N* = 58	11–13 ^6/7^ weeks *N* = 598	≥14 weeks *N* = 63	*p* - Value
Age (years)	33.9 ± 4.5	34.0 ± 5.2	34.2 ± 5.4	0.96
** BMI (kg/m ^2^ ) ** a	**24.1 ± 4.6**	**27.9 ± 6.3**	**28.1 ± 7.4**	**<0.001**
Parity	1.0 (0–2.0)	1.0 (0–1.0)	1.0 (0–2.0)	0.35
Race				0.31
American Indian/Alaska native	1 (1.7%)	9 (1.5%)	1 (1.6%)	–
Asian	2 (3.4%)	46 (8.3%)	5 (8.1%)	
Black or African American	1 (1.7%)	34 (6.1%)	3 (4.8%)	
Other	3 (5.1%)	17 (3.1%)	4 (6.4%)	
Unknown/not reported	7 (12.1%)	81 (13.5%)	14 (22.2%)	
White	44 (75.9%)	411 (73.9%)	36 (58.1%)	
Ethnicity				0.11
Hispanic or Latino	3 (5.2%)	99 (17.2%)	8 (12.7%)	–
Not Hispanic or Latino	50 (86.2%)	443 (77.0%)	46 (73.0%)	
Unknown/not reported	5 (8.6%)	33 (5.7%)	9 (14.3%)	
Spontaneous pregnancy	45/50 (90.0%)	362/390 (92.8%)	41/47 (87.2%)	0.35
Tobacco use	0 (0%)	1 (0.3%)	0 (0%)	0.92
Maternal comorbidities				
Chronic hypertension	1 (2.0%)	11 (2.8%)	2 (4.3%)	0.79
Diabetes (type 1 and 2)	0 (0%)	9 (2.3%)	0 (0%)	0.32
Autoimmune disease	2 (4.0%)	17 (4.4%)	2 (4.3%)	0.99
Cardiac disease	0 (0%)	7 (1.8%)	1 (2.1%)	0.62
Thyroid disease	7 (14.0%)	53 (13.6%)	6 (12.8%)	0.98
Psychiatric history	5 (8.6%)	21 (3.5%)	1 (1.5%)	0.09

Data are presented as mean ± standard deviation, median (interquartile range), *n* (%), and *n* / *N* (%). BMI, body mass index.

a Bold text signifies statistical significance *p* <0.05.

Early CVS was more frequently performed transvaginally (58.6% vs. 27.5% vs. 22.2%, *p* < 0.001, Table 2 ). Early CVS was often indicated for genetic history (63.8% vs. 26.8% vs. 6.3%, *p* < 0.001) and less often for abnormal serum screening (3.4% vs. 40.0% vs. 76.2%, *p* < 0.001) or abnormal ultrasound findings (20.7% vs. 43.0% vs. 44.6%, *p* = 0.004). The number of passes and the placental location were similar between groups. The proportion of abnormal karyotype results was lowest in the early CVS group (12.1% vs. 37.8% vs. 46.8%, *p* < 0.001). Similarly, abnormal microarray results were least frequent following early CVS (2.0% vs. 21.4% vs. 29.4%, *p* = 0.002). Procedure-related complications were rare and not statistically different between groups. No limb abnormalities were reported.

**Table 2:** Chorionic villus sampling variables among early, typical, and late gestational ages

	<11 weeks *N* = 58	11–13 ^6/7^ weeks *N* = 598	≥14 weeks *N* = 63	*p* - Value
**Transvaginal route**	**34 (58.6%)**	**164 (27.5%)**	**14 (22.2%)**	**<0.001**
Number of passes	1 (1–1)	1 (1–1)	1 (1–1)	0.15
Placenta location				0.46
Anterior	27 (46.6%)	277 (46.6%)	34 (54.0%)	–
Bilobed	0 (0%)	1 (0.2%)	0 (0%)	
Fundal	1 (1.7%)	43 (7.2%)	2 (3.2%)	
Lateral	1 (1.7%)	33 (5.6%)	2 (3.2%)	
Posterior	29 (50.0%)	240 (40.4%)	25 (39.7%)	
Indication for CVS (percentage)				
** Abnormal antenatal serum screening**	**2 (3.4%)**	**239 (40.0%)**	**48 (76.2%)**	**<0.001**
** Abnormal ultrasound findings**	**12 (20.7%)**	**257 (43.0%)**	**28 (44.4%)**	**0.004**
** Genetic history** a	**37 (63.8%)**	**160 (26.8%)**	**4 (6.3%)**	**<0.001**
Other b	12 (20.7%)	145 (24.2%)	17 (27.0%)	0.72
**Total abnormal karyotype results**	**7/58 (12.1%)**	**221/585 (37.8%)**	**29/62 (46.8%)**	**<0.001**
**Total abnormal microarray results**	**1/50 (2.0%)**	**86/402 (21.4%)**	**10/34 (29.4%)**	**0.002**
CVS complications c	0 (0%)	4 (0.7%)	0 (0%)	> 0.9

Abbreviation: CVS, chorionic villus sampling.

Data are presented as *n* (percentage), and *n* / *N* (percentage). Bold values signifies statistical significance *p* <0.05.

a Genetic history includes maternal carrier status, personal or family history of genetic anomalies, and/or prior pregnancies with genetic anomalies.

b Other includes AMA and maternal anxiety or preference.

c CVS complications include miscarriage, bleeding, infection, or limb abnormalities.

The primary outcome of median CVS tissue weight obtained was comparable among the three groups and across all participating sites (12 vs. 15 vs. 15 mg, *p* = 0.35, Table 3 ). When evaluating secondary outcomes, the need for culture did not differ significantly between groups, regardless of abnormal microarray result ( Table 4 ). Time to microarray result was shortest in the typical group (9 vs. 8 vs. 10 days, *p* = 0.046), though the observed difference of 1 to 2 days is small and unlikely to be clinically meaningful. Time to result for the normal microarray was similar. Adequate sample collection, defined as ≥5 mg, was achieved in over 89% of procedures in all groups, with no statistically significant differences stratified by gestational age ( Table 4 ).

**Table 3:** Primary outcome among early, typical, and late gestational ages

	<11 weeks *N* = 58	11–13 ^6/7^ weeks *N* = 595	≥14 weeks *N* = 62	*p* -Value
Chorionic villi weight (mg)	12 (10–15)	15 (8–30)	15 (8–24.75)	0.35
NYU Langone LI ( *n* = 444)	15 (10–18.75)	10 (5–20)	10 (6–20)	0.39
Colorado ( *n* = 232)	36.5 (27.25–40)	33 (20–45)	40 (26.25–48.5)	0.72
Sinai West ( *n* = 43)	10 (10–13.5)	–	10 (8–13.5)	0.57

Data are presented as median (interquartile range).

**Table 4:** Secondary outcomes among early, typical, and late gestational ages

	<11 weeks *N* = 50	11–13 ^6/7^ weeks *N* = 400	≥14 weeks *N* = 34	*p* -Value
Need for culture	7 (14.0%)	87 (21.8%)	10 (29.4%)	0.08
Normal microarray	7/49 (14.3%)	67/308 (21.8%)	7/24 (29.2%)	0.31
Abnormal microarray	0/1 (0%)	20/92 (21.7%)	3/10 (30%)	–
**Length of time (days)**	**9 (7–14)**	**8 (7–13)**	**10 (9–14)**	**0.041**
Normal microarray	9 (7–14)	8 (7–13)	11 (8.5–16)	0.09
Abnormal microarray	14	9 (6–12)	10 (9–11.5)	–
Adequate sample (≥5 mg)	56/58 (96.6%)	533/598 (89.1%)	60/63 (95.2%)	0.07
Termination of pregnancy	7/41 (17.1%)	156/491 (31.8%)	22/57 (38.6%)	0.07
Spontaneous abortion	1/40 (2.5%)	24/434 (5.5%)	1/41 (2.4%)	0.51

Data are presented as median (interquartile range), *n* (%), and *n* / *N* (%).

The rate of TOP trended higher in the late CVS group, though not statistically significant (17.1% vs. 31.8% vs. 38.6%, *p* = 0.07). Among ongoing pregnancies, the mean gestational age at delivery was similar between groups (38–39 weeks, *p* = 0.26). Early and late CVS was not associated with increased risk of preterm birth, gestational diabetes, or composite placental insufficiency as compared with the typical group ( Table 5 ).

**Table 5:** Obstetric outcomes and risk ratio among early, typical, and late gestational ages

	<11 weeks *n* = 41	*p* -Value	11–13 ^6/7^ weeks *n* = 338	≥14 weeks *n* = 32	*p* - Value
Gestational age at delivery (weeks)	39.0 ± 1.2	–	38.4 ± 1.9	38.1 ± 2.3	0.26
Gestational diabetes	0.62 (0.14–2.81)	0.54	–	1.02 (0.33–3.18)	0.97
Composite placental insufficiency a	0.68 (0.20–2.29)	0.53	–	1.16 (0.43–3.12)	0.77

Data are presented as mean ± standard deviation, and median (interquartile range).

a Composite placental insufficiency includes fetal growth restriction, abruption, oligohydramnios, and preeclampsia with severe features.

## Discussion

This multicenter, retrospective study evaluated the technical feasibility of CVS outside the typical gestational window by comparing villi yield and diagnostic performance. The primary outcome of median CV weight did not differ among groups and exceeded the adequate ≥5 mg threshold, indicating that both early and late CVS can yield sufficient villi for reliable genetic analysis.

Although the time to microarray was statistically different, the absolute difference (1–2 days) was small and unlikely to be clinically significant. Moreover, microarray results were available prior to the option for amniocentesis at 16 weeks. Some may consider early amniocentesis; however, DNA yield is limited and often necessitates culture with an additional 10- to 14-day turnaround time. 11 12 In this study, the need for culture did not differ among groups, supporting that adequate tissue was obtained regardless of gestational age.

Early CVS was more commonly performed transvaginally. Empirically, early placentas were often easily accessible vaginally. Transabdominal CVS may be more technically challenging in earlier gestations with a smaller uterus and less developed placenta. 13 Further, early CVS was more common for genetic history rather than abnormal screening, reflective of clinical practice. Patients with a known genetic condition or prior affected pregnancy frequently pursue diagnostic testing early, whereas abnormal screening results typically arise later, prompting diagnostic procedures during the typical or late CVS window.

Our study specifically evaluated early CVS between 10 and 11 weeks. Although current recommendations consider this interval within the standard of care, some providers remain hesitant to perform CVS before 11 weeks. Rather, patients often opt for non-invasive testing with cfDNA during this time. With advances in cfDNA testing and carrier screening technologies, some companies are offering Non-Invasive Prenatal Testing (NIPT) as early as 8 to 9 weeks, and patients may seek earlier diagnostic confirmation. This study demonstrates that early CVS can provide adequate tissue for evaluation. Usual caution needs to be applied when choosing the best follow-up diagnostic testing after high-risk NIPT results, as certain aneuploidies are more likely to result in confined placental mosaicism. 14

Conversely, the initiative for earlier anatomical surveys at 13 to 14 weeks may increase the desire for diagnostic testing. The 14- to 16-week window represents a diagnostic gap during which neither CVS nor amniocentesis is typically available. Our results suggest the feasibility of CVS to provide adequate tissue for microarray without the need to wait for an amniocentesis. Providers can then facilitate timely counseling and pregnancy management, potentially reducing morbidity and mortality associated with later second-trimester terminations.

The presented analysis did not include non-viable pregnancies at the time of CVS. However, on initial data collection, 15 patients underwent CVS for the indication of fetal demise, with 6 patients with non-viable pregnancies between 7 and 9 weeks' gestation. Inclusion of these patients in a separate analysis resulted in a similar conclusion with appropriate villi yield without prolonged time to culture. This supports the utilization of early CVS for genetic evaluation in early pregnancy loss when non-procedural management would preclude tissue analysis. However, inclusion does not inform safety in ongoing pregnancies and should not be interpreted as such at earlier gestations.

While complication rates, including miscarriage, bleeding, and infection up to 4 weeks postprocedure, were low and similar between groups, the study was not powered to evaluate safety. No limb anomalies were reported, though CVS was not performed in viable pregnancies <10 weeks. This aligns with contemporary data suggesting that this complication is rare with improved training and technique. 15 There were also no differences in obstetric outcomes, including gestational age at delivery, gestational diabetes, and a composite of placental insufficiency (fetal growth restriction, placental abruption, oligohydramnios, and preeclampsia). These findings support the procedural feasibility of CVS across the evaluated gestational windows without evidence of adverse pregnancy outcomes. This analysis focused on technical feasibility and diagnostic adequacy rather than procedural safety, which warrants further evaluation in prospective studies.

Strengths include the evaluation of the largest U.S. cohort across three academic, high-volume CVS centers with varying operative experience. Early and late CVS was performed across all institutions by both MFM attendings and fellows with varying clinical experience from 1 to >20 years of practice. The multicenter design enhances generalizability, reflecting routine variation in operator experience and cytogenetic processing between institutions.

Limitations include intersite variability in reported villi weights, which is an estimation and may vary by operator and cytogenetic laboratory. Despite the use of standard photographic weight references, interlaboratory variability likely contributed to observed differences in reported villi weight. 16 Nevertheless, all sites consistently exceeded adequate tissue yields (≥5 mg), and there were no differences among the groups at each site individually. Typical CVS data were not collected from one center (Carnegie), though the large typical cohort mitigates this balance. Further, delivery outcomes were limited by variations in follow-up and patient care sites. Despite these limitations, the findings strongly support the procedural feasibility of early and late CVS without evidence of reduced yield or higher complication rates.

## Conclusion

In this large, multicenter cohort, both early (<11 weeks) and late (≥14 weeks) CVS achieved CV yields, diagnostic adequacy, and pregnancy outcomes comparable to the traditional 11 ^0/7^ - to 13 ^6/7^ -week window. Tissue adequacy exceeded 89% across all groups, and complications remained low. These findings challenge the conventional limits of CVS timing from a technical perspective. This also supports the utilization of early CVS for cytogenetic testing in early pregnancy loss, where medical management may not allow for direct tissue testing. Prospective studies evaluating safety and long-term outcomes are warranted to further inform clinical practice.
